# Sentiment Analysis of Tweets on Menu Labeling Regulations in the US

**DOI:** 10.3390/nu15194269

**Published:** 2023-10-06

**Authors:** Yuyi Yang, Nan Lin, Quinlan Batcheller, Qianzi Zhou, Jami Anderson, Ruopeng An

**Affiliations:** 1 Division of Computational and Data Science, Washington University, St. Louis, MO 63130, USA; 2 Brown School, Washington University, St. Louis, MO 63130, USA; b.quinlan@wustl.edu; 3 Department of Statistics and Data Science, Washington University, St. Louis, MO 63130, USA; nlin@wustl.edu; 4 Department of Molecular Microbiology, Washington University School of Medicine, St. Louis, MO 63110, USA; qianzi@wustl.edu; 5 Implementation Science Center for Cancer Control, Washington University, St. Louis, MO 63130, USA; ajami@wustl.edu

**Keywords:** menu labeling, calorie counts, sentiment analysis, Twitter, public health policy, obesity, deep learning

## Abstract

Menu labeling regulations in the United States mandate chain restaurants to display calorie information for standard menu items, intending to facilitate healthy dietary choices and address obesity concerns. For this study, we utilized machine learning techniques to conduct a novel sentiment analysis of public opinions regarding menu labeling regulations, drawing on Twitter data from 2008 to 2022. Tweets were collected through a systematic search strategy and annotated as positive, negative, neutral, or news. Our temporal analysis revealed that tweeting peaked around major policy announcements, with a majority categorized as neutral or news-related. The prevalence of news tweets declined after 2017, as neutral views became more common over time. Deep neural network models like RoBERTa achieved strong performance (92% accuracy) in classifying sentiments. Key predictors of tweet sentiments identified by the random forest model included the author’s followers and tweeting activity. Despite limitations such as Twitter’s demographic biases, our analysis provides unique insights into the evolution of perceptions on the regulations since their inception, including the recent rise in negative sentiment. It underscores social media’s utility for continuously monitoring public attitudes to inform health policy development, execution, and refinement.

## 1. Introduction

The United States of America (US) has experienced a sharp increase in the prevalence of obesity over the past several decades. An estimated 41.9% of US adults were categorized as obese based on the National Health and Nutrition Examination Survey (NHANES) 2017–2020, a significant increase from 30.5% in 1999–2000 [1,2]. In addition to posing substantial risks through chronic conditions like type 2 diabetes and cardiovascular disease, the complications associated with obesity in COVID-19 cases were underscored throughout the pandemic [3,4]. This shed light on shifting national perspectives and ignited discussions about obesity’s impact, exploring potential strategies to enhance both individual and public health [3,4]. 

Researchers have considered a pathway involving the examination of individual posts on social media, particularly focusing on sentiment analyses to explore dietary and health perspectives among individual users and commercial advertisers across various platforms [5]. Sentiment analysis is the computational study of people’s opinions, sentiments, emotions, and attitudes toward various subjects [6]. With the ubiquity of social media, it has become an invaluable tool to gauge public opinion on a myriad of topics, including public policies [7,8]. Using social media data for sentiment analysis offers advantages over traditional surveying techniques. First, it provides access to a vast and diverse audience in real-time, enabling the continuous monitoring of public sentiment as it evolves [9]. Additionally, the spontaneous nature of social media posts may capture more candid and immediate responses compared to pre-structured surveys [10]. However, potential biases can arise from the demographic representation in specific social platforms, and the often informal and succinct nature of posts can lead to challenges in accurately capturing nuanced opinions [11]. Despite these challenges, the prevalence of social media data, coupled with advancements in artificial intelligence and computational capabilities, has revolutionized sentiment analysis [12]. Advanced algorithms now facilitate efficient text mining [12], offering a dynamic lens through which we can view and assess the public’s perspective on pivotal policies like menu labeling regulations.

A study from the Pew Research Center indicated that among major social media platforms, Twitter is the most frequently used platform among journalists within the US, and it ranks among the top three platforms in terms of public users [5]. Since its inception in the mid-2000s, Twitter has become a forum for expressing, studying, and potentially modifying health behaviors [13,14,15]. Results from a systematic review indicated that Twitter can be used in health research to explore content, context, sentiment, and engagement with health information [15]. In addition, results from a tweet content analysis suggested that advertising companies utilized Twitter during the COVID-19 pandemic to influence food and beverage selections [14]. Thus, Twitter is an ideal resource to explore population-level perspectives regarding policies and views that may affect weight-related behaviors and outcomes in the US [8,15].

The World Health Organization (WHO) recommends implementing population-level policies that promote healthier diet adoption [16]. Previous studies have revealed the profound impact of contextual factors such as neighborhood food access, environment, government policies, and the COVID-19 pandemic on individuals’ dietary intake, physical activity, and obesity [17,18]. The menu labeling regulation in the US, commonly called the “calorie counting law,” is a foundation policy in public health nutrition [19]. Instituted as part of the Affordable Care Act of 2010 (Section 4205), chain restaurants and similar retail food establishments with 20 or more locations are required to prominently display calorie information for standard menu items [20]. The primary intent of this policy is to provide consumers with transparent nutritional information, facilitating informed dietary choices [21]. In 2014, the Food and Drug Administration (FDA) further clarified the requirements to include three components restaurants must display or provide (1) calorie counts of standard menu items, (2) a written post stating that an average daily caloric intake is 2000 calories, and (3) a written post noting that additional detailed nutritional information is available upon request at the restaurant [22]. By enhancing consumer awareness, it aims to address the growing concerns of overeating and its subsequent health ramifications, including the obesity epidemic [23].

Therefore, the current study aims to undertake a comprehensive sentiment analysis of menu labeling regulations by assessing tweets posted from 2008 to 2022. This timeframe incorporates the implementation of local-level menu labeling laws in 2008 (e.g., New York City’s 2008 menu labeling mandate), national menu labeling law establishment in 2010 and enactment in 2014, and the COVID-19 pandemic from 2019 to 2022 [14,22,24]. In addition, this timeframe largely coincides with Twitter’s increasing popularity as a forum for national conversations from 2006 to 2022 [25]. 

Several features distinguish our research from prior studies. First, to our knowledge, this study represents the inaugural exploration of public sentiment toward menu labeling regulations via social media data. Second, we employed the Twitter application programming interface (API) for academic research, amassing a dataset of 7253 pertinent tweets through a systematic search strategy. Third, our methodology involved using manually annotated data to design a deep neural network model in the field of natural language processing (NLP), enabling the autonomous classification of sentiments in future tweets concerning menu labeling regulations. Last, our analysis chronologically evaluates the evolution of public sentiments and perceptions, identifying pivotal factors influencing these sentiments. The study’s findings are anticipated to offer valuable insights for designing, implementing, and refining menu labeling regulations, supporting their broader acceptance and minimizing potential misperceptions.

## 2. Methods

### 2.1. Data Collection, Annotation, and Analysis

Using the Twitter API for academic research, we constructed and implemented a search algorithm to systematically identify and collect tweets about menu labeling regulations. The search algorithm consists of a comprehensive list of keywords (e.g., “calorie counts”) and hashtags (e.g., “#menulabeling”) concerning menu labeling (Appendix A). The search was limited to tweets written in English, and retweets were excluded. We retrieved 7253 tweets posted from 1 January 2008 to 10 December 2022. Python programs and related open-source APIs were used for data retrieval (e.g., Tweepy) and modeling (e.g., Hugging Face Transformers).

We annotated all tweets using four predetermined, mutually exclusive categories, namely “positive,” “negative,” “neutral,” and “news.” The former three refer to the sentiment in the tweet text, whereas “news” denotes a tweet of an excerpt or web link to a news article without revealing the author’s sentiment. As an initial step, three co-authors, Y.Y., Q.B., and Q.Z., independently annotated 200 randomly selected tweets from the pool. They then resolved discrepancies through discussion, from which they developed a coherent understanding of sentiment classification. The three co-authors subsequently annotated the remaining tweets independently and compared the results. The following rules were adopted to finalize the sentiment of a tweet: if two or all three co-authors assigned the same sentiment to a tweet, that sentiment was auto-assigned to the tweet without discussion; if each co-author assigned a different sentiment to a tweet, a discussion was held among the three co-authors to make a joint decision. The interrater reliability among the three co-authors was measured by the intra-class correlation coefficient (0.88). Doccano, a Python-based, open-source text annotation tool, was used to annotate tweets.

We calculated the prevalence of the four sentiments and tracked their annual trends from 2008 to 2022. Additionally, we employed a random forest model to assess the relationship between five covariates (i.e., number of followers, number of tweets an author posted, number of retweets, number of replies, and number of likes) and tweet sentiments. Feature importance is a common machine learning technique that calculates and assigns a score to each input feature (i.e., covariate) based on its usefulness in predicting a target (e.g., tweet sentiment). We calculated the standardized feature importance score (bounded between 0 and 1, with a higher score denoting more importance) from the random forest model.

We utilized the train_test_split function from the scikit-learn library to randomly split the 7253 tweets into training, validation, and test sets consisting of 80% (5801 tweets), 10% (726), and 10% (726), respectively. We tokenized individual tweets and their assigned sentiments in the three sets before feeding them to the NLP models. The training and validation sets were used to train and evaluate the models, respectively, whereas the test set was reserved for assessing model performance.

### 2.2. NLP Model Building

We performed transfer learning using the tokenized training set to fine-tune the NLP models. Transfer learning is a powerful technique that enables knowledge obtained from solving one problem to be applied to a different but related problem [26]. For example, knowledge obtained while learning to predict the next word in Wikipedia documents, stored as trainable weights in a deep neural network model, may be used when building a different model to categorize scientific journal abstracts. This study employed three large, state-of-the-art NLP models—RoBERTa, XLM-RoBERTa, and Twitter-XLM-RoBERTa—to classify tweets into one of the four sentiment categories. RoBERTa is a transformer model that was pre-trained on a vast corpus of English text in a self-supervised fashion. XLM-RoBERTa is a multilingual version of RoBERTa that was pre-trained on text data written in 100 languages. Twitter-XLM-RoBERTa is a specialized version of XLM-RoBERTa that has been fine-tuned on nearly 200 million multilingual tweets. Although the study concentrated solely on English-language tweets, the multilingual models proved to leverage the similarities and complex relations between multiple languages to improve performances [27]. Each model was initialized with its pre-trained weights. We fine-tuned each model for multiple epochs until the validation loss stabilized, upon which we stopped fine-tuning and chose the model checkpoint with the lowest validation loss. The performances of the three models were further assessed on the test set.

We performed text augmentation to rebalance the distribution of sentiment categories. Sentiment categories were highly unbalanced in the original dataset—the “news” category occupies over three-quarters, whereas the “positive” and “negative” categories were less than 4%, respectively. Text augmentation is a commonly applied technique to replenish rarer categories by generating variations of the original text while preserving their intent. Thus, we employed two text augmentation strategies, synonym and embedding, to balance the sentiment categories in the training and validation set. In the synonym augmentation, multiple tweet variations were generated by replacing some of the words in the original tweet with their synonyms. In the embedding augmentation, multiple tweet variations were generated by modifying the embeddings (i.e., numerical representations of words in an NLP model) used to represent the words in the original tweet. Following text augmentation, the training and validation sets had an equal number of tweets in all four sentiment categories.

The three models were fine-tuned on the initial training and validation sets and their augmented counterparts. Model performances on the test sets were compared using accuracy and F1 score.

This study involved the use of public tweet data and did not involve any human or animal subjects; therefore, it was exempted from the human subjects review regulations by the Washington University’s Institutional Review Board.

## 3. Results

**Figure 1:**
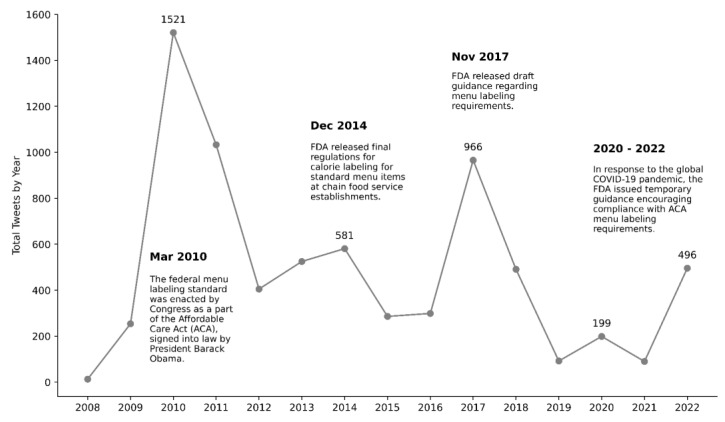
Annual number of menu labeling regulation-related tweets (2008–2022).

Using the Twitter API for academic research, we identified and downloaded 7253 tweets related to menu labeling regulations posted between 1 January 2008 and 10 December 2022. Figure 1 depicts the annual number of tweets, along with significant events concerning menu labeling regulations. In 2008, a modest 13 tweets addressed this topic. This count experienced a sharp rise in 2009, culminating in a record 1521 tweets in 2010. This spike aligns with the anticipation and implementation of local-level mandates in 2008 and the ultimate implementation of the national-level menu labeling standard in March 2010 [24]. After this peak, there was a marked decrease, with numbers falling by nearly 75% in 2012. In the ensuing years, the volume varied, with another pronounced rise to around 1000 tweets in 2017. This surge corresponds to the Food and Drug Administration’s (FDA) issuance of draft guidance on menu labeling protocols in November of the same year. After 2017, a sharp decline ensued, reaching a low of under 100 tweets by 2019. Notably, the data indicate a rebound to almost 500 tweets in 2022, which can be associated with the FDA’s interim guidance in 2020 during the COVID-19 pandemic, indicating a revitalized public engagement with menu labeling norms.

Among all tweets collected, over three-quarters were categorized as “news,” and less than a fifth were categorized as “neutral,” whereas the “positive” and “negative” categories each occupied roughly 3.6%. Here, we will provide examples for each category. For instance, a “positive” tweet supported the legislation: “Should pass a law that all restaurants have to post calorie counts on items. Americans deserve to know what they are eating @BarackObama.” Conversely, a “negative” tweet argued against the legislation: “You are wrong in the fact that even people with healthy eating habits can be triggered into disordered eating, and counting calories is just one way of that showing up. It should not be a law [but] it should simply be an option to access the calorie count of foods.” A “neutral” tweet expressed curiosity about the potential impact of the legislation: “I am wondering if the new law in CA (California), to have the calorie count on the menu board, will change people’s eating habits.” Finally, a “news” tweet reported on a delay in the implementation of calorie count laws: “Delay Likely for US Calorie Count Law [web link to the news].” The results suggest a dominance of news-related tweets about calorie count laws, with relatively few expressing either positive or negative sentiments.

**Figure 2:**
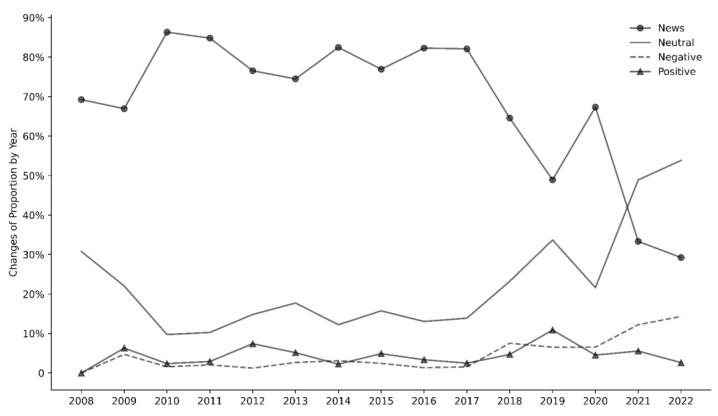
Prevalence of menu labeling regulation-related tweet sentiments (News, Neutral, Negative, or Positive) from 2008 to 2022.

Figure 2 illustrates the temporal trends in the prevalence of “news,” “neutral,” “negative,” and “positive” tweets from 2008 to 2022. A sharp decline in the prevalence of “news” tweets was observed after 2017, with the highest peak occurring in 2010 and 2011 (86%); this dropped significantly to 29% in 2021 and 2022. Conversely, a marked increase in the prevalence of “neutral” tweets is evident after 2017. Initially, the prevalence of “neutral” tweets fell by two-thirds from 2008 to 2011 (10%) and remained relatively stable (15%) until 2017 before rising sharply to its highest level in 2022 (54%). By contrast, the prevalence of “negative” tweets remained low but steadily increased from 2017 onwards, reaching its highest level in 2022 (14%). Similarly, the prevalence of “positive” tweets remained low and relatively stable over the years, peaking in 2019 (11%). When excluding “news” tweets, the proportions of “neutral,” “negative,” and “positive” sentiment tweets between 2008 and 2022 counted for 70.8%, 14.5%, and 14.6%, respectively.

**Figure 3:**
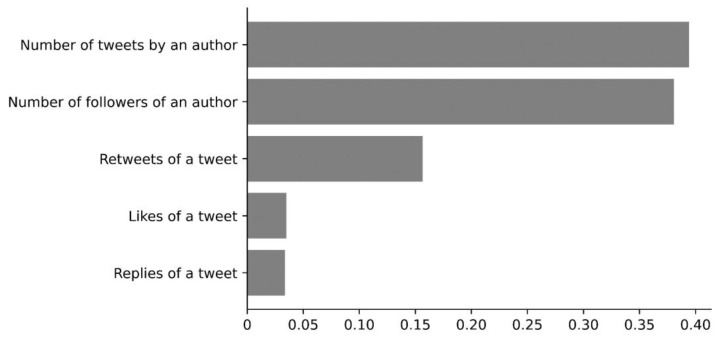
Correlates with menu labeling regulation-related tweet sentiments.

We employed a random forest model to evaluate the covariates associated with the four tweet categories. Figure 3 presents the standardized feature importance scores for the covariates under investigation. Our findings reveal that an author’s total number of tweets carried the highest importance score (0.39), followed closely by the number of an author’s followers (0.38) and retweets (0.16). In contrast, the importance scores for the number of likes (0.03) and replies (0.03) were considerably lower. This analysis underscores the significance of an author’s overall Twitter activity and degree of influence while suggesting that likes and replies may have less bearing on the results.

Table 1 presents the accuracy and F1 scores for the three aforementioned pre-trained language models (RoBERTa, XLM-RoBERTa, and Twitter-XLM-RoBERTa) at various epochs on the test set. The performances of these models were relatively similar, with accuracy rates between 88% and 92% and F1 scores ranging from 86% to 91%. RoBERTa emerged as the top performer, achieving an accuracy of 91.5% and an F1 score of 91.1%.

Table 2 presents the accuracy and F1 scores of the same pre-trained models on the identical test set after incorporating two text augmentation strategies (synonym and embedding) during training. Before augmentation, Twitter-XLM-RoBERTa performed the worst among the three models. However, its performance improved post-augmentation, showing a 2.4% increase in accuracy, a 1.7% increase in F1 score with synonym augmentation, and a 1.7% rise in both accuracy and F1 score with embedding augmentation. By contrast, the other two models experienced a slight decline in performance after augmentation, indicating that text augmentation may not be universally helpful. This discrepancy could result from differences in model architecture, hyperparameters, and potential noise or bias introduced by augmentation.

## 4. Discussion

This study conducted a sentiment analysis of public opinions regarding menu labeling regulations in the US using Twitter data from 2008 to 2022. The analysis found that the volume of relevant tweets peaked around major policy announcements related to menu labeling, underscoring Twitter’s utility for gauging public engagement. The majority of tweets were categorized as neutral or news-related, with relatively few expressing outright positive or negative sentiments. The prevalence of news tweets declined sharply after 2017, while neutral views became more prevalent over time. Several tweet features, like the author’s followers and past activity, proved informative for predicting sentiment categories. It is worth noting that while our results align with other studies suggesting consistency in an author’s sentiment over multiple posts [28], our study did not specifically analyze the relationship between these factors and the sentiment of individual tweets (positive/neutral/negative). Additional analyses would be needed to explore this concept and determine causality fully.

Overall, our natural language processing models achieved strong performance in classifying tweet sentiments, aided by text augmentation techniques in select cases. Overall, this novel social media-based analysis provided intriguing insights into the evolution of public perceptions on menu labeling laws since their inception. In our analysis of the sentiment categories, we found that the majority of the tweets collected were categorized as “news.” When “news” tweets were excluded from our analysis, the proportions of “neutral,” “negative,” and “positive” tweets between 2008 and 2022 stood at 70.8%, 14.5%, and 14.6%, respectively. The predominance of “news” tweets suggests that the Twitter discourse around menu labeling is mainly informational, often serving as a platform for disseminating updates on legislative developments or delays in implementation. In addition, the temporal patterns in sentiment categories offer valuable insights into the shifting nature of public discourse on menu labeling regulations. The prevalence of “news” tweets sharply declined after 2017, from 86% in 2010 to 29% in 2022.

In contrast, “neutral” tweets increased significantly after 2017, reaching 54% in 2022. The marked decline in the proportion of “news” tweets and the corresponding increase in “neutral” tweets could indicate a shift from informational to more exploratory or contemplative discussions among the public. While the prevalence of “negative” tweets remained relatively low, there was a steady rise from 2017, peaking at 14% in 2022. This suggests a growing segment of the population may be expressing concerns or criticisms regarding menu labeling laws. The “positive” tweets fluctuated less, peaking modestly at 11% in 2019. This relatively stable proportion, on the other hand, indicates a consistent, albeit small, group of proponents. In addition, given the overlapping timeframe with the COVID-19 pandemic, it is possible that these variations also reflect shifting perspectives associated with the unanticipated food-related challenges of the COVID-19 pandemic that may have influenced public sentiment of population-level policies, such as food shortages, increasing prices, and interruptions in the food supply chain [29]. These findings point to a complex and evolving landscape of public sentiment toward menu labeling regulations.

Despite its powerful capacity to influence public sentiment and drive social change, social media is still largely untapped as a resource for informing governmental decisions and policy initiatives [30]. Various avenues exist for integrating social media into health policy development and execution. These include gauging public perceptions and attitudes toward specific policies, shaping societal norms and opinions, identifying geographic or demographic groups disproportionately affected by policy outcomes, and fostering social campaigns that either support or challenge existing regulations [31]. The real-time monitoring of social media platforms can help to uncover crucial societal trends, such as shifts in sentiment, policy popularity, sources of misinformation, and divisions arising from policy changes [32]. However, analyses of social media data have historically been limited by computational constraints and the lack of effective algorithms. Notable challenges include the massive scale of the data, the unstructured nature of the data (which can consist of text, images, videos, and audio), and data quality issues such as typos and low-resolution media. Traditional statistical methods often fall short when applied to unstructured data [33]. However, recent advancements in deep learning techniques, like convolutional neural networks for image analysis and NLP transformers, coupled with increased computing power through graphics processing units (GPUs) and cloud technology, have made the real-time analysis of large-scale social media data feasible. This study, which intends to inform policy and legislative actions, showcases the utility and promise of leveraging AI-based models for extracting meaningful insights from extensive social media datasets. This study took approximately three months to conduct and involved generating a database of tweets referencing menu labeling laws or mandates and building an NLP model to categorize tweet sentiments and the other elements noted in this manuscript. The timeframe to complete this study is considerably shorter than traditional methods. In addition, the trained model can be applied to classify future tweets by their sentiments in an automatic fashion. However, periodically retraining the model is likely needed due to potential data drafting (e.g., new patterns emerging from future tweets that the original model has not learned). Considering that many political movements and legislative actions require rapidly produced and analyzed evidence, the reduced timeframe of research and evaluation involving AI-based models is promising to support evidence-based legislation. However, additional research is necessary to fully explore the value of AI-based models to support policy and legislative actions.

While traditional research on the advantages and disadvantages of menu labeling regulations often relies on data from structured surveys, interviews, and transaction logs from food establishments [34,35], these methods may not fully encompass the public’s diverse views, attitudes on the subject, and complexities interjected by the COVID-19 pandemic. Although data-driven decision-making is essential, it should be complemented by a nuanced understanding of public sentiment and timeframes. Our study addresses this gap by conducting a sentiment analysis of tweets spanning more than a decade, introducing a fresh perspective to the policy-making discourse. We observed that public attention to menu labeling regulations, as measured by tweet volume, hit its highest point in 2010. As the topic became less novel, the majority of the tweets shifted from a news-based to a neutral sentiment. Notably, since 2020, tweets expressing negative sentiment have begun to outnumber those with a positive outlook, although both remain relatively infrequent.

Further analysis, such as topic modeling or thematic analysis, is warranted to grasp the nuances of these sentiments. Our preliminary findings from the tweets suggest that opposition to menu labeling often centers around themes like personal freedoms, the impact on small businesses, and concerns about triggering unhealthy eating behaviors. These insights offer valuable guidance for lawmakers considering similar regulations in different jurisdictions and for current policymakers contemplating adjustments or public awareness campaigns related to existing menu labeling laws.

The sentiment expressed in tweets about menu labeling was found to correlate with the authors’ overall Twitter activity, number of followers, and the extent of retweets. The cross-sectional, observational approach limits our ability to make causal inferences. However, our modeling results suggested that more influential Twitter users—those with a high tweet count and a large follower base—are more inclined to share their views on menu labeling policies. This observation aligns with our previous research on soda tax sentiment analysis, where we found that authors with a large cohort of followers were more likely to express their perceptions and attitudes toward soda taxes [36]. These findings underscore the role of influential figures in shaping public opinion on policy matters. Studies have consistently shown that social networks exhibit power imbalances, and the advent of social media influencers has the potential to amplify these disparities [37]. As such, these individuals may disproportionately impact public sentiment toward health policies. Recognizing the influence of these key players can offer insights into how public opinion is formed and manipulated, which is essential for policymakers seeking to gauge public support for specific legislative initiatives.

In this study, we fine-tuned three NLP models to categorize sentiments in tweets about menu labeling. Initially, all three models demonstrated comparable performance without text augmentation, with accuracy rates ranging from 88% to 92% and F1 scores between 86% and 91%. RoBERTa was the superior model in terms of performance, attaining an accuracy of 91.5% and an F1 score of 91.1%. An interesting pattern emerged after implementing text augmentation techniques, namely the synonym and embedding augmentations. Twitter-XLM-RoBERTa, which initially underperformed, exhibited marked improvement in both accuracy and F1 score.

Conversely, the performance of RoBERTa and XLM-RoBERTa slightly declined after the text augmentation, indicating that such techniques are not universally beneficial across all models. These variations could be attributed to differences in model architecture, hyperparameters, and the potential introduction of noise and bias through the augmentation methods. This emphasizes the need for a tailored approach when applying text augmentation, considering each model’s specific attributes and requirements. Despite the improvements, Twitter-XLM-RoBERTa did not surpass the performance of RoBERTa without augmentation. Consequently, while text augmentation can enhance a model’s generalizability, its effectiveness is dependent on its unique characteristics and the training data. Therefore, text augmentation should be considered one of several strategies, along with model selection and hyperparameter tuning, to optimize machine learning model performance.

The several limitations in the scope and methodology of this study are highlighted as follows: First, Twitter users tend to skew younger and may not represent the broader US population, limiting the generalizability of our study findings [38]. Second, the lack of reliable geographic metadata restricts our capacity to perform a comparative sentiment analysis between people residing in areas with and without menu labeling regulations. Third, Twitter’s character limitations—140 characters before 8 November 2017, and 280 characters after that—could restrict the nuance and complexity of the opinions expressed with respect to menu labeling. Future research could incorporate data from other social media platforms, such as Facebook, Instagram, and Reddit, to obtain a fuller picture of public sentiment. Fourth, although we used state-of-the-art NLP models for sentiment classification, the results were not error-free. A larger, annotated dataset would be ideal for training more accurate models, but such an endeavor was beyond the resources available for this study. Finally, while sentiment analysis provides a valuable overview of public opinion on menu labeling, more nuanced insights could be gleaned from additional qualitative analyses, such as thematic or topic modeling.

In summary, this study examined public sentiment on menu labeling regulations in the US, leveraging systematically collected Twitter data and advanced deep neural network models. The public’s focus on menu labeling, as evidenced by the annual volume of relevant tweets, peaked in 2010 and has since shown fluctuations. Remarkably, a decline in the proportion of tweets solely disseminating news about menu labeling coincided with an increase in the proportion expressing neutral sentiment toward these regulations. Starting in 2020, tweets bearing a negative sentiment have outnumbered those with a positive sentiment, although both remain relatively low in proportion. Excluding news-related tweets, the sentiment distribution from 2008 to 2022 is predominantly neutral, standing at 70.8%, followed by positive and negative sentiments at 14.6% and 14.5%, respectively. This study also identified several predictors of tweet sentiment, including the author’s overall tweet activity, follower count, and retweet metrics. While additional research is needed to explore these findings and other findings within our study more thoroughly, these insights may be valuable for shaping the development, implementation, and modification of menu labeling policies and enhancing public comprehension and support while reducing potential misunderstandings.

## Figures and Tables

**Table 1:** Model performances in test set (before augmentation).

Model	Epochs	Accuracy	F1 Score
RoBERTa	1	0.901	0.902
	2	0.915	0.911
	3	0.912	0.908
XLM-RoBERTa	3	0.875	0.856
	4	0.899	0.896
	5	0.880	0.877
Twitter-XLM-RoBERTa	1	0.882	0.880
	2	0.883	0.880
	3	0.882	0.880

**Table 2:** Model performances in test set (after augmentation).

Model	Augmentation Strategy	Epochs	Accuracy	F1 Score
RoBERTa	synonym	2	0.910	0.910
	embedding	2	0.895	0.896
XLM-RoBERTa	synonym	4	0.893	0.888
	embedding	4	0.891	0.890
Twitter-XLM-RoBERTa	synonym	2	0.904	0.895
	embedding	2	0.898	0.895

## Data Availability

The search algorithm is shown in Appendix A.

## Appendix A

Search Algorithm in Twitter API for Academic Research

“menu labelling” OR “menu labeling” OR “calorie labelling” OR “calorie labeling” OR “nutrition labelling” OR “nutrition labeling” OR “calorie count” OR “calorie counts” OR #menulabelling OR #menulabeling OR #calorielabelling OR #calorielabeling OR #nutritionlabelling OR #nutritionlabeling OR #caloriecount OR #caloriecounts OR #caloriecountlaw # caloriecountslaw OR (#menu #labelling) OR (#menu #labeling) OR (#calorie #labelling) OR (#calorie #labeling) OR (#nutrition #labelling) OR (#nutrition #labeling) OR (#calorie #count) OR (#calorie #counts) lang:en -is:retweet

## References

[1] Rakhra V., Galappaththy S.L., Bulchandani S., Cabandugama P.K. (2020). Obesity and the western diet: How we got here. Mo Med..

[2] Stierman B., Afful J., Carroll M.D., Chen T.-C., Davy O., Fink S., Fryar C.D., Gu Q., Hales C.M., Hughes J.P. (2021). National Health and Nutrition Examination Survey 2017–March 2020 Prepandemic Data Files Development of Files and Prevalence Estimates for Selected Health Outcomes. https://stacks.cdc.gov/view/cdc/106273.

[3] Scully T., Ettela A., LeRoith D., Gallagher E.J. (2021). Obesity, type 2 diabetes, and cancer risk. Front. Oncol..

[4] Kissin R., Khoury L., Wallenborn G., Kothari S.N. (2023). When the COVID-19 pandemic collides with the obesity epidemic in the United States: A national survey. Surg. Obes. Relat. Dis..

[5] Jurkowitz M., Gottfried J. (2022). Twitter Is the Go-to Social Media Site for U.S. Journalists, but Not for the Public. Pew Research Center. https://www.pewresearch.org/fact-tank/2022/06/27/twitter-is-the-go-to-social-media-site-for-u-s-journalists-but-not-for-the-public.

[6] Liu B. (2020). Sentiment Analysis: Mining Opinions, Sentiments, and Emotions.

[7] Sobkowicz P., Kaschesky M., Bouchard G. (2012). Opinion mining in social media: Modeling, simulating, and forecasting political opinions in the web. Gov. Inf. Q..

[8] Bartlett C., Wurtz R. (2015). Twitter and Public Health. J. Public Health Manag. Pract..

[9] Anstead N., O’Loughlin B. (2015). Social media analysis and public opinion: The 2010 UK general election. J. Comput.-Mediat. Commun..

[10] Tamersoy A., De Choudhury M., Chau D.H. Characterizing smoking and drinking abstinence from social media. Proceedings of the 26th ACM Conference on Hypertext & Social Media.

[11] Tucker J.A., Guess A., Barberá P., Vaccari C., Siegel A., Sanovich S., Stukal D., Nyhan B. (2018). Social Media, Political Polarization, and Political Disinformation: A Review of the Scientific Literature.

[12] Pozzi F.A., Fersini E., Messina E., Liu B. (2016). Sentiment Analysis in Social Networks.

[13] Song J., Jin D.-L., Song T.M., Lee S.H. (2023). Exploring Future Signals of COVID-19 and Response to Information Diffusion Using Social Media Big Data. Int. J. Environ. Res. Public Health.

[14] Tsai K.A., Cassidy O., Arshonsky J., Bond S., Del Giudice I.M., Bragg M.A. (2022). COVID-washing in US food and beverage marketing on Twitter: Content analysis. JMIR Form. Res..

[15] Sinnenberg L., Buttenheim A.M., Padrez K., Mancheno C., Ungar L., Merchant R.M. (2017). Twitter as a Tool for Health Research: A Systematic Review. Am. J. Public Health.

[16] World Health Organization Obesity and Overweight. https://www.who.int/en/news-room/fact-sheets/detail/obesity-and-overweight.

[17] Dixon B.N., Ugwoaba U.A., Brockmann A.N., Ross K.M. (2021). Associations between the built environment and dietary intake, physical activity, and obesity: A scoping review of reviews. Obes. Rev..

[18] Stefan N., Birkenfeld A.L., Schulze M.B. (2021). Global pandemics interconnected—Obesity, impaired metabolic health and COVID-19. Nat. Rev. Endocrinol..

[19] Moghimi E., Wiktorowicz M.E. (2019). Regulating the Fast-Food Landscape: Canadian News Media Representation of the Healthy Menu Choices Act. Int. J. Environ. Res. Public Health.

[20] Stein K. (2010). A national approach to restaurant menu labeling: The Patient Protection and Affordable Health Care Act, Section 4205. J. Am. Diet. Assoc..

[21] Kelly B., Jewell J. (2018). What Is the Evidence on the Policy Specifications, Development Processes and Effectiveness of Existing Front-of-Pack Food Labelling Policies in the WHO European Region?.

[22] Goldman T.R. (2015). The FDA’s Menu-Labeling Rule (Updated). https://www.healthaffairs.org/do/10.1377/hpb20150713.56602.

[23] McGeown L. (2019). The calorie counter-intuitive effect of restaurant menu calorie labelling. Can. J. Public Health.

[24] Restrepo B., Minor T. (2018). New National Menu Labeling Provides Information Consumers Can Use to Help Manage Their Calorie Intake. https://www.ers.usda.gov/amber-waves/2018/october/new-national-menu-labeling-provides-information-consumers-can-use-to-help-manage-their-calorie-intake.

[25] Vanian J. (2022). Twitter Is Now Owned by Elon Musk—Here’s a Brief History from the App’s Founding in 2006 to the Present. CNBC. https://www.cnbc.com/2022/10/29/a-brief-history-of-twitter-from-its-founding-in-2006-to-musk-takeover.html.

[26] Yang Q., Zhang Y., Dai W., Pan S.J. (2020). Transfer Learning.

[27] Goyal V., Kumar S., Sharma D.M. Efficient neural machine translation for low-resource languages via exploiting related languages. Proceedings of the 58th Annual Meeting of the Association for Computational Linguistics: Student Research Workshop.

[28] Zou X., Yang J., Zhang J. (2018). Microblog sentiment analysis using social and topic context. PLoS ONE.

[29] Eskandari F., Lake A., Butler M. (2022). COVID-19 pandemic and food poverty conversations: Social network analysis of twitter data. Nutr. Bull..

[30] Khan G.F. (2017). Social Media for Government.

[31] Bou-Karroum L., El-Jardali F., Hemadi N., Faraj Y., Ojha U., Shahrour M., Darzi A., Ali M., Doumit C., Langlois E.V. (2017). Using media to impact health policy-making: An integrative systematic review. Implement. Sci..

[32] Rowe F., Mahony M., Graells-Garrido E., Rango M., Sievers N. (2021). Using Twitter to track immigration sentiment during early stages of the COVID-19 pandemic. Data Policy.

[33] Rajula H.S.R., Verlato G., Manchia M., Antonucci N., Fanos V. (2020). Comparison of Conventional Statistical Methods with Machine Learning in Medicine: Diagnosis, Drug Development, and Treatment. Medicina.

[34] Patel P.C., Struckell E.M., Ojha D. (2020). Calorie labeling law and fast food chain performance: The value of capital responsiveness under sales volatility. J. Bus. Res..

[35] Vasiljevic M., Cartwright E., Pilling M., Lee M.-M., Bignardi G., Pechey R., Hollands G.J., Jebb S.A., Marteau T.M. (2018). Impact of calorie labelling in worksite cafeterias: A stepped wedge randomised controlled pilot trial. Int. J. Behav. Nutr. Phys. Act..

[36] An R., Yang Y., Batcheller Q., Zhou Q. (2023). Sentiment Analysis of Tweets on Soda Taxes. J. Public Health Manag. Pract..

[37] Casero-Ripollés A. (2021). Influencers in the Political Conversation on Twitter: Identifying Digital Authority with Big Data. Sustainability.

[38] Morgan-Lopez A.A., Kim A.E., Chew R.F., Ruddle P. (2017). Predicting Age Groups of Twitter Users Based on Language and Metadata Features. PLoS ONE.

